# Stepping inside the whispers and tingles: multisensory virtual reality for enhanced relaxation and wellbeing

**DOI:** 10.3389/fdgth.2023.1212586

**Published:** 2023-07-18

**Authors:** Ina Kaleva, Simon Riches

**Affiliations:** ^1^ Department of Psychosis Studies, Institute of Psychiatry, Psychology & Neuroscience, King’s College London, London, United Kingdom; ^2^ South London and Maudsley NHS Foundation Trust, London, United Kingdom; ^3^ Department of Psychology, Institute of Psychiatry, Psychology & Neuroscience, King’s College London, London, United Kingdom; ^4^ Social, Genetic and Developmental Psychiatry Centre, Institute of Psychiatry, Psychology & Neuroscience, King’s College London, London, United Kingdom

**Keywords:** autonomous sensory meridian response, ASMR, virtual reality, relaxation, mental health, stress reduction, psychological wellbeing, internet

## Introduction

Although traditional relaxation practices, such as mindfulness, meditation, and yoga, can be effective to increase positive wellbeing and reduce stress, individuals display inconsistent adherence to these activities because they can be time-consuming and difficult to sustain (1–4). These adherence issues emphasise the need for novel, accessible relaxation and stress-reduction interventions to support positive mental health. Within the growing digital mental health and health-tech research, there is increasing evidence of the health and wellbeing benefits of social media use and technology-mediated interventions (5, 6). The aim of the present article is to describe how two recent, popular technological developments, virtual reality (VR) relaxation and autonomous sensory meridian response (ASMR) online videos, can be combined to provide enhanced relaxation and wellbeing interventions suitable for busy, modern lives. VR-based ASMR interventions can be especially accessible to active social media users, such as younger people (7); however, VR-based ASMR has the potential to be enjoyed by people of all ages and all levels of competence with technology.

## Virtual reality relaxation and autonomous sensory meridian response

VR relaxation shows promising results to support positive wellbeing in both the general population and clinical samples (8, 9). It primarily involves users viewing calming nature scenes, such as beaches, mountains, and forests, using a head-mounted display that provides audio and visuals in a three-dimensional format. In contrast to traditional, cognitively taxing relaxation practices that can involve memory and imagination, VR immerses users in vivid and realistic imagery and sound in a convenient and accessible way (10). ASMR, on the other hand, is a pleasant tingling body sensation and feeling of wellness in response to visual and audio stimuli, known as “triggers”, and it has been largely associated with online videos. Popular videos dedicated to inducing ASMR include common audio triggers, such as whispering, mouth sounds, fingernail tapping, brushing, scratching, nature, and water sounds, as well as visual triggers, such as gentle hand movements, simulated face touching, eye contact, and close-up camera proximity. ASMR videos have a similar function to VR, by offering immersive stimuli that induce real feelings and bodily sensations, which create the experience of “being there” inside the virtual or video environment. Since immersion is so key to their impact, the next step in the development of these technologies is to combine VR and ASMR. VR-based ASMR has the potential to be more immersive than VR alone or two-dimensional (2D) screen ASMR while providing a powerful combination of immersive audio-visual stimulation.

ASMR videos have become an online phenomenon on YouTube and TikTok, attracting millions of viewers and subscribers. A popular type of ASMR video is personal attention roleplay, in which the focus is on the viewer, with such videos reaching nearly 50 million views on YouTube. In personal attention roleplay, ASMR content creators (“ASMRtists”) often role-play make-up artists brushing viewers’ faces, hairdressers carrying out scalp massages, or physicians performing medical examinations (often eye, ear, or cranial nerve). Personal attention ASMR videos often have clear and emphasised visual triggers (e.g., eye contact and gentle hand movements) and whispered positive affirmations and comfort. Some viewers prefer “no talking” videos, consisting only of triggers like mouth sounds, blowing, crinkling, scratching, brushing, and tapping. Popular YouTube channels of ASMRtists are approaching 21 million subscribers. Some of the most popular videos of all time include eating (known as “Mukbang”) (11), medical examinations (12), haircutting and whispering (13), mouth sounds (14), and scratching and tapping (15). A YouTube search by authors in March 2023 showed that the most viewed ASMR video on YouTube, with over 437 million streams, is a Mukbang video of eating sweets and desserts (16). Popular TikTok accounts are approaching six million followers. A TikTok search by authors in March 2023 showed that videos containing the ASMR hashtag (#ASMR) have a total of 631 billion views. An exhibition on this topic, called *Weird Sensation Feels Good: The World of ASMR*, was launched in the UK during Mental Health Awareness Week in May 2022, featuring immersive and interactive ASMR installations, and it ran again in April 2023.

ASMR can have deeply relaxing and sedating effects while working as a distraction from negative thoughts. Research indicates it can provide temporary relief to anxiety, depression, insomnia, and chronic pain (17–19). A functional MRI study found that ASMR experience is significantly associated with brain areas involved in emotional arousal, satisfaction, and reward (20). On a physiological level, it has been found to reduce heart rate (an indicator of calmness) and increase skin conductance levels (an indicator of excitement), suggesting that ASMR is a relaxing but also an exciting and stimulating experience (21). As a result of the attention that ASMR receives from people experiencing mental health problems, ASMRtists have designed specific, therapeutic ASMR content tailored to this population, which aims to induce relief from symptoms. For example, videos aiming to relieve anxiety or panic often contain whispered soothing affirmations (22, 23), as well as gentle movements pretending to “brush away” or “pluck away” viewer's tension (24), and videos designed for people experiencing insomnia are specifically dedicated to facilitating sleep (25).

## Complementary technologies

New 360° and VR180 formats of ASMR videos, suitable to experience in VR head-mounted displays, are emerging (26), which could enhance visual stimulation and immersion. VR technology is designed to produce a visual experience very similar to how the human eye perceives the real world (27), whereas contemporary ASMR videos tend to use binaural audio, a technique for recording sounds with two or more microphones, creating a targeted ear-to-ear audio experience (28). Combining the distinct immersive features of ASMR videos and VR can lead to important advances in these two technological developments resulting in powerful complementary multisensory stimulation with a potential therapeutic effect. Creating ASMR content in a VR format has the potential to dramatically increase the perceived proximity and realism of visual triggers allowing the viewer to “step inside” the ASMR video, while satisfying the need of VR to be more multisensory. This could enhance the sense of presence in the ASMR video, which in turn could intensify the perceived bodily sensations. Such immersion could also enrich the experience of emotional and physical intimacy, which is an important element of personal attention roleplay. Adding binaural ASMR audio to the VR environment provides an opportunity to better localise sounds, which could further enhance realism and provide a fully immersive soundscape. The use of audio in VR plays a crucial role in perceived realism and user satisfaction, yet it is still undervalued and lacks consistent research (29).

Although the idea of VR-based ASMR has gained mainstream popularity in the last few years, it has not received much scientific attention and is largely unstudied by academic researchers. To date, only a few studies have explored this new concept (30). One study indicated that adding ASMR audio to VR can make the experience slightly more immersive, compared to standard stereo audio, in a sample of junior college students (31). Another study implemented ASMR, along with other sensory cues, within VR and showed this can stimulate multisensory sexual arousal in a sample of 140 adults (32). There is also a scientific poster that proposes a study design for implementing a VR-based method to induce ASMR using light triggers (33). Existing research has focused more on the general effects of combining VR and ASMR in adults and young people with the aim of improving authenticity, sense of presence, immersion, and overall VR and ASMR experiences. There is a lack of empirical studies on the feasibility and effectiveness of VR-based ASMR specifically for the purpose of enhancing relaxation and wellbeing.

VR-based ASMR could have useful practical and research implications to enhance wellbeing. Given that VR and ASMR are both safe and accessible, a hybrid approach could work as a feasible and cost-effective home- or work-based intervention for wellbeing support and stress management in the general population, especially for younger people who actively use social media platforms, such as TikTok and YouTube (7). Research has indicated the benefits of nature-based VR relaxation in reducing burnout and stress in a range of workforces, including healthcare professionals and other key workers (34–37). According to Attention Restoration Theory, natural environments support individuals in achieving emotional separation from their concerns, which boosts mental fatigue recovery (38). Adding binaural ASMR audio capturing soothing nature sounds, such as beach waves or wind blowing through the trees, could supplement this experience inducing a state of deeper relaxation. Adding therapeutic personal attention roleplay elements to the calming nature-based virtual scenes of VR relaxation could also make the experience more engaging, relaxing, and stimulating for optimal wellbeing. For example, creating the experience of having one's hair brushed while relaxing on a virtual beach, rather than experiencing this on a 2D screen, could increase the benefits of the intervention.

## Clinical applications

Given the acceptability of both VR relaxation and ASMR to people experiencing mental health problems (8, 18, 19, 39), there is a huge potential for the therapeutic and clinical applications of VR-based ASMR, which could use positive affirmations and coping techniques or guided meditation exercises, among other techniques. With meditation practices, gentle whispering could contribute to the experience of comfort and safety, while immersive VR environments could support individuals in focusing attention on the present moment (40). These interventions could be especially effective for people with high levels of stress, anxiety-related conditions, insomnia, or chronic physical pain. VR-based ASMR could provide a high degree of flexibility and personalisation by offering the option to choose between a range of different triggers depending on the preferences of the individual.

Merging VR and ASMR will create opportunities for novel clinical applications. ASMRtists have already started to create content tailored to specific mental health conditions that is mostly enjoyed by viewers in non-clinical settings. In the future, ASMRtists may consider working in collaboration or consultation with mental health professionals to design VR-based ASMR content that is safe to deliver to clinical populations. Moreover, ASMRtists should consider patient involvement in the planning and designing of their VR content to ensure that the content is tailored to their needs. This could improve the effectiveness of these interventions and address ethical considerations related to potential risks.

Older adults tend to be less likely to use social media and may face obstacles to using technology (41). This may lead to barriers to accessing VR-based ASMR and prevent them from experiencing the potential benefits of these interventions. Strategies to narrow the digital divide and increase the accessibility of VR-based ASMR for people of all ages and backgrounds could be applied. Although VR-based ASMR is popular on YouTube, which is a commonly used platform by a wider age group, ASMRtists could engage more actively with the Facebook audience as this is the largest platform used by people of a wide range of ages (7). Enhancing age diversity among ASMRtists may be another way to increase accessibility, given that age similarity between the viewer and the ASMRtist could offer a greater connection and sense of belonging (42).

## Conclusion

VR relaxation and ASMR are promising and complementary interventions. The combination of VR and ASMR to enhance relaxation and wellbeing is largely unstudied by research, and the scientific basis of ASMR and its effect on mental health is still very limited. Given the promising evidence for VR relaxation, more research is needed to investigate the potential benefits of VR-based ASMR.

## References

[1] Anderson T Suresh M Farb NA. Meditation benefits and drawbacks: empirical codebook and implications for teaching. J Cogn Enhanc. (2019) 3:207–20. 10.1007/s41465-018-00119-y

[2] Cagas JY Biddle SJH Vergeer I. When an activity is more than just exercise: a scoping review of facilitators and barriers for yoga participation. Int Rev Sport Exerc Psychol. (2020) 1–62. 10.1080/1750984X.2020.1827448

[3] Koszycki D Danilewitz M Maclean H Sanchez-Campos M Gonsalves C Archibald D Feasibility and effectiveness of an online mindfulness meditation program for medical students. Can Med Educ J. (2018) 9:e15–e25. 10.36834/cmej.43041 30498540 PMC6260511

[4] Farias M Wikholm C. Has the science of mindfulness lost its mind? BJPsych Bull. (2016) 40:329–32. 10.1192/pb.bp.116.053686 28377813 PMC5353526

[5] Brown G Rathbone AL Prescott J. Social media use for supporting mental health (SMILE). Ment Heal Rev J. (2021) 26:279–97. 10.1108/MHRJ-10-2020-0079

[6] Fu S Fang J Cai Z Lim ETK Tan CW Yang H. Advancing health-related abilities and behaviors via health apps: a large-scale survey from self-regulation perspective. Internet Res. (2022) 32:1097–130. 10.1108/INTR-09-2020-0485

[7] Pew Research Center. Social Media Use in 2021. (2021). p. 1–4. Available at: www.pewresearch.org

[8] Riches S Jeyarajaguru P Taylor L Fialho C Little J Ahmed L Virtual reality relaxation for people with mental health conditions: a systematic review. Soc Psychiatry Psychiatr Epidemiol. (2023) 58:989–1007 10.1007/s00127-022-02417-5 36658261 PMC9852806

[9] Riches S Azevedo L Bird L Pisani S Valmaggia L. Virtual reality relaxation for the general population: a systematic review. Soc Psychiatry Psychiatr Epidemiol. (2021) 56:1707–27. 10.1007/s00127-021-02110-z 34120220 PMC8197783

[10] Slater M Gonzalez-Liencres C Haggard P Vinkers C Gregory-Clarke R Jelley S The ethics of realism in virtual and augmented reality. Front Virtual Real. (2020) 1:1. 10.3389/frvir.2020.00001

[11] Zach Choi. Most popular food for ASMR with Stephanie Soo (Honeycomb, Aloe Vera, Tanghulu, Macarons). (2019) Available at: https://www.youtube.com/watch?v=VNnZ5H3SmS4&t=333s (Accessed May 12, 2023).

[12] Gibi ASMR. Fastest ASMR | Dentist, Eye, Cranial Nerve, Sleep Clinic, Lice, Ear Exam, Ear Cleaning, Makeup, Spa! (2021). Available at: https://www.youtube.com/watch?v=EEF0pg9hSpQ&t=636s (Accessed May 12, 2023).

[13] Gentle Whispering ASMR. Sleep-inducing Haircut ASMR | Shampoo | Page Flipping | Scissors. (2018). Available at: https://www.youtube.com/watch?v=gf_MqDBBMPI (Accessed May 12, 2023).

[14] FrivolousFox ASMR. ASMR Ear Licking∼Extreme Mouth Sounds for Tingle Immunity. (2018). Available at: https://www.youtube.com/watch?v=-BdSCcmJKJk&t=733s (Accessed May 12, 2023).

[15] ASMR Glow. ASMR 50 Triggers for ∼2H of Tingles (Fluffy Ears, Mic Scratching, Plastic Cups, Pop Rocks +). (2019). Available at: https://www.youtube.com/watch?v=Tt7Tk0FUyBw&t=4322s (Accessed May 12, 2023).

[16] Hongyu ASMR 홍유. ASMR Rainbow Desserts 무지개 먹방 팝핑보바, 보석젤리, 별사탕 먹방 Popping Boba, Jelly, Gummy Mukbang No Talking Eating. (2020). Available at: https://www.youtube.com/watch?v=mzN_x5hWtlU (Accessed November 27, 2022).

[17] Barratt EL Davis NJ. Autonomous sensory meridian response (ASMR): a flow-like mental state. PeerJ (2015) 3:e851. 10.7717/peerj.851 25834771 PMC4380153

[18] Hu MQ Li HL Huang SQ Jin YT Wang SS Ying L Reduction of psychological cravings and anxiety in women compulsorily isolated for detoxification using autonomous sensory meridian response (ASMR). Brain Behav. (2022) 12:e2636. 10.1002/brb3.2636 35674485 PMC9304838

[19] Smejka T Wiggs L. The effects of autonomous sensory meridian response (ASMR) videos on arousal and mood in adults with and without depression and insomnia. J Affect Disord. (2022) 301:60–7. 10.1016/j.jad.2021.12.015 34915083

[20] Lochte BC Guillory SA Richard CAH Kelley WM. An fMRI investigation of the neural correlates underlying the autonomous sensory meridian response (ASMR). BioImpacts. (2018) 8:295–304. 10.15171/bi.2018.32 30397584 PMC6209833

[21] Poerio GL Blakey E Hostler TJ Veltri T. More than a feeling: autonomous sensory meridian response (ASMR) is characterized by reliable changes in affect and physiology. PLoS One. (2018) 13:e0196645. 10.1371/journal.pone.0196645 29924796 PMC6010208

[22] Sarah Lavender ASMR. ASMR for ANXIETY and PANIC Relief|Helping you calm down. (2022). Available at: https://www.youtube.com/watch?v=CM_ZDGorTn8&ab_channel=SarahLavenderASMR (Accessed November 27, 2022).

[23] softlygaloshes ASMR. [BINAURAL ASMR] Emotional Breakdown Relief (ear to ear whispering). (2015). Available at: https://www.youtube.com/watch?v=GRbpy2SjAHA&ab_channel=softlygaloshesASMR (Accessed November 27, 2022).

[24] Gibi ASMR. 4 K HD ASMR|Plucking Away Anxieties. (2019). Available at: https://www.youtube.com/watch?v=Ito3WvJT__g&t=100s&ab_channel=GibiASMR (Accessed November 27, 2022).

[25] ASMR Glow. ASMR Pulling Insomnia Out of You. (2022). Available at: https://www.youtube.com/watch?v=KHTWDbRwL6c&ab_channel=ASMRGlow (Accessed November 27, 2022).

[26] VR Lisa ASMR. Super Realistic Tingly Haircut in VR! | ASMR. (2022). Available at: https://www.youtube.com/watch?v=9l_BCX_sp-U (Accessed November 27, 2022).

[27] Creem-Regehr SH Stefanucci JK Bodenheimer B. Perceiving distance in virtual reality: theoretical insights from contemporary technologies. Philos Trans R Soc B Biol Sci. (2023) 378:20210456. 10.1098/rstb.2021.0456 PMC9745869 36511405

[28] Geronazzo M Spagnol S Avanzini F. Mixed structural modeling of head-related transfer functions for customized binaural audio delivery. 18th International Conference on Digital Signal Processing (DSP); Fira, Greece (2013). 10.1109/ICDSP.2013.6622764

[29] de Villiers Bosman I Buruk OO Jørgensen K Hamari J. The effect of audio on the experience in virtual reality: a scoping review. Behav Inf Technol. (2023) 1–35. 10.1080/0144929X.2022.2158371

[30] Klausen HB. The ambiguity of technology in ASMR experiences: four types of intimacies and struggles in the user comments on YouTube. Nord Rev. (2021) 42:124–36. 10.2478/nor-2021-0045

[31] Chung S-M Chen Z-Y Wu C-T. Synaesthesia sound design in virtual reality. In: Mobasheri A, editor. ArtsIT 2022 lecture notes of the institute for computer sciences, social informatics and telecommunications engineering. Vol. 479. Cham: Springer (2023). p. 535–41.

[32] Aleksandrovich A Gomes LM. Shared multisensory sexual arousal in virtual reality (VR) environments. *Paladyn**,* J Behav Robot (2020) 11:379–89. 10.1515/pjbr-2020-0018

[33] Peng D Pai Y Minamizawa K. asmVR: light triggers in virtual reality to induce ASMR. International Conference on Artificial Reality and Telexistence Eurographics Symposium on Virtual Environments. (2022) 10.2312/egve.20221295

[34] Morse G Salyers MP Rollins AL Monroe-DeVita M Pfahler C. Burnout in mental health services: a review of the problem and its remediation. Adm Policy Ment Health. (2012) 39:341–52. 10.1007/s10488-011-0352-1 21533847 PMC3156844

[35] Riches S Smith H. Editorial: taking a break in the “new normal”: virtual reality relaxation for a stressed workforce. Ment Health Rev J. (2022) 27:133–6. 10.1108/MHRJ-06-2022-095

[36] Riches S Taylor L Jeyarajaguru P Veling W Valmaggia L. Virtual reality and immersive technologies to promote workplace wellbeing: a systematic review. J Ment Health. (2023):1–21. 10.1080/09638237.2023.2182428 36919828

[37] Stetz MC Kaloi-Chen JY Turner DD Bouchard S Riva G Wiederhold BK. The effectiveness of technology-enhanced relaxation techniques for military medical warriors. Mil Med. (2011) 176:1065–70. 10.7205/MILMED-D-10-00393 21987967

[38] Berto R. The role of nature in coping with psycho-physiological stress: a literature review on restorativeness. Behav Sci. (2014) 4:394–409. 10.3390/bs4040394 25431444 PMC4287696

[39] Veling W Lestestuiver B Jongma M Hoenders HJR Van DC. Virtual reality relaxation for patients with a psychiatric disorder: crossover randomized controlled trial. J Med Internet Res. (2021) 23:e17233. 10.2196/17233 33448933 PMC7846446

[40] Seabrook E Kelly R Foley F Theiler S Thomas N Wadley G Understanding how virtual reality can support mindfulness practice: mixed methods study. J Med Internet Res. (2020) 22:e16106. 10.2196/16106 32186519 PMC7113800

[41] Moore RC Hancock JT. Older adults, social technologies, and the coronavirus pandemic: challenges, strengths, and strategies for support. Soc Media Soc. (2020) 6:2056305120948162. 10.1177/2056305120948162

[42] Rosen DC Miller AB Nakash O Halpern L Alegría M. Interpersonal complementarity in the mental health intake: a mixed-methods study. J Couns Psychol. (2012) 59:185–96. 10.1037/a0027045 22268575 PMC6338422

